# The experience of transcatheter closure of postoperative ventricular septal defect after total correction

**DOI:** 10.1186/s13019-019-0933-8

**Published:** 2019-06-11

**Authors:** N’goran Yves N’da Kouakou, Jinyoung Song, June Huh, I-Seok Kang

**Affiliations:** 1 Department of Cardiology, Institute Cardiology d’Abidjan, Abidjan, Côte d’Ivoire; 2 Department of Pediatrics, Samsung Medical Center, Heart Vascular Stroke Institute, Sungkyunkwan University School of Medicine, 81 Irwon-ro, Gangnam-gu, Seoul, 06351 South Korea

**Keywords:** Catheterization, Heart septal defects, Ventricles, Postoperative

## Abstract

**Background:**

The purpose of this study was to describe our experience with patients who underwent transcatheter closure of a post-operative ventricular septal defect (VSD).

**Methods:**

All patients who underwent transcatheter closure of a VSD after total correction of congenital heart disease since 2012 were enrolled. Medical records were retrospectively reviewed to determine the patients’ initial diagnosis, closure device used, and final outcome after device closure.

**Results:**

Six patients with a median age of 17.7 years (range: 7 months–48 years) underwent transcatheter closure of an unresolved VSD. The median time interval from the initial corrective surgery to the percutaneous closure procedure was 10.4 years (range: 0.3–33.0 years). The initial diagnoses included tetralogy of Fallot (one patient), VSD (two patients), double outlet of the right ventricle (two patients), and aortic valve stenosis (one patient). The reasons for unresolved VSD (other than leakage) after corrective surgery included previous fenestration (in two patients), and iatrogenic Gerbode shunt (in one patient). Various devices were used, including the Amplatzer duct occluder I, Amplatzer duct occluder II, Amplatzer vascular plug II, and Cocoon membranous VSD occluder. Only one device was used in each patient. There were no major complications associated with the closure procedures. The immediate results were satisfactory. The median follow-up duration was 2.75 years. All cases were successful, with the exception of minimal leak in one patient.

**Conclusions:**

Transcatheter device closure of post-operative VSD can be performed using various device types of devices and is safe and effective. But more experiences are mandatory.

## Background

Postoperative ventricular septal defect (VSD) is a relatively common finding after correction of an isolated VSD or complex cardiac anomalies. Postoperative VSD may arise due to patch dehiscence, suture disruption, incomplete closure of the defect, or bacterial endocarditis. The incidence of postoperative VSD varies according to the type of initial defect, and ranges from 5 to 25% [1]. Although some of these residual VSDs are restrictive and well tolerated, they may result in left-to-right shunting, persistent left ventricular volume overload, or pulmonary hypertension. Therefore, postoperative VSDs may require re-intervention [2]. Rarely, an iatrogenic VSD may form after surgery. VSD closure with fenestration is another cause of postoperative VSD. Surgical repair remains the mainstay of treatment for postoperative residual VSD. A repeat surgical repair procedure (involving cardiopulmonary bypass and sternotomy) is physically and psychologically traumatic for pediatric patients given the requirement for extracorporeal circulation and risks of myocardial scarring and bleeding [3]. Transcatheter closure of a post-operative VSD has emerged as a less invasive approach that can be used in selected patients to avoid the high morbidity and mortality associated with repeated surgical repair [4].

However, few studies have evaluated the transcatheter approach in the treatment of postoperative VSD [5, 6]. Therefore**,** we reviewed our institutional experience with transcatheter closure of postoperative residual VSDs and report mid-term outcomes.

## Methods

Six patients who underwent transcatheter closure of postoperative VSDs at Samsung Medical Center between January 2012 and December 2017 were enrolled. Patients’ medical records were reviewed retrospectively. Transthoracic echocardiography (TTE) was performed preoperatively to evaluate the VSD size and location, potential hemodynamic consequences, and any associated lesions prior to attempting a postoperative VSD repair. The indications for transcatheter closure of postoperative VSDs included a VSD with clinical evidence of volume overload, or pulmonary hypertension due to shunt. Device closure can be considered when the VSD is adequately separated in space from adjacent cardiac structures, including the aortic and tricuspid valves.

The procedure was performed under general anesthesia and using the guidance of transesophageal echocardiography (TEE). Patients received 100 IU/kg intravenous heparin immediately after the femoral artery was accessed. Continuous heparin infusion was maintained to reach an activated clotting time target of 200 msec. Following routine cardiac catheterization and hemodynamic evaluation, left ventricular angiographic images were obtained to determine the most appropriate device for closure.

Similar procedures were applied in every patient. The VSD was crossed in a retrograde fashion from the aorta using a 0.035″ Terumo® guide wire, which was snared at the pulmonary artery and withdrawn through the femoral venous sheath (femoral arteriovenous loop). After ensuring that the tricuspid chordae had not been crossed, the catheter was advanced over the guide wire to the inferior vena cava. A long delivery sheath was passed over the guide wire from the femoral vein to the ascending aorta or left ventricle. The device was then attached to the delivery cable and passed through the delivery sheath. The distal disc was opened in the aorta or the left ventricle, and then the entire system was withdrawn. After confirming that the left ventricular disc was in the correct position, the other portion of the device was opened in the right ventricle. After verifying the position of the device using TEE and angiography, the device was released. We did not attempt any cases without the femoral arteriovenous loop.

After device implantation, a continuous intravenous heparin infusion was administered. Electrocardiographic monitoring was performed for 24 h after closure. If there were no complications, the patient was discharged 2 days later. Aspirin was prescribed at 3–5 mg/kg/day for 6 months following the procedure. A TTE was performed on the first postoperative day, and then regularly at 3, 6, and 12 months in the outpatient clinic.

The institutional review board approved this study. The need for informed consent was waived.

## Results

**Fig. 1:**
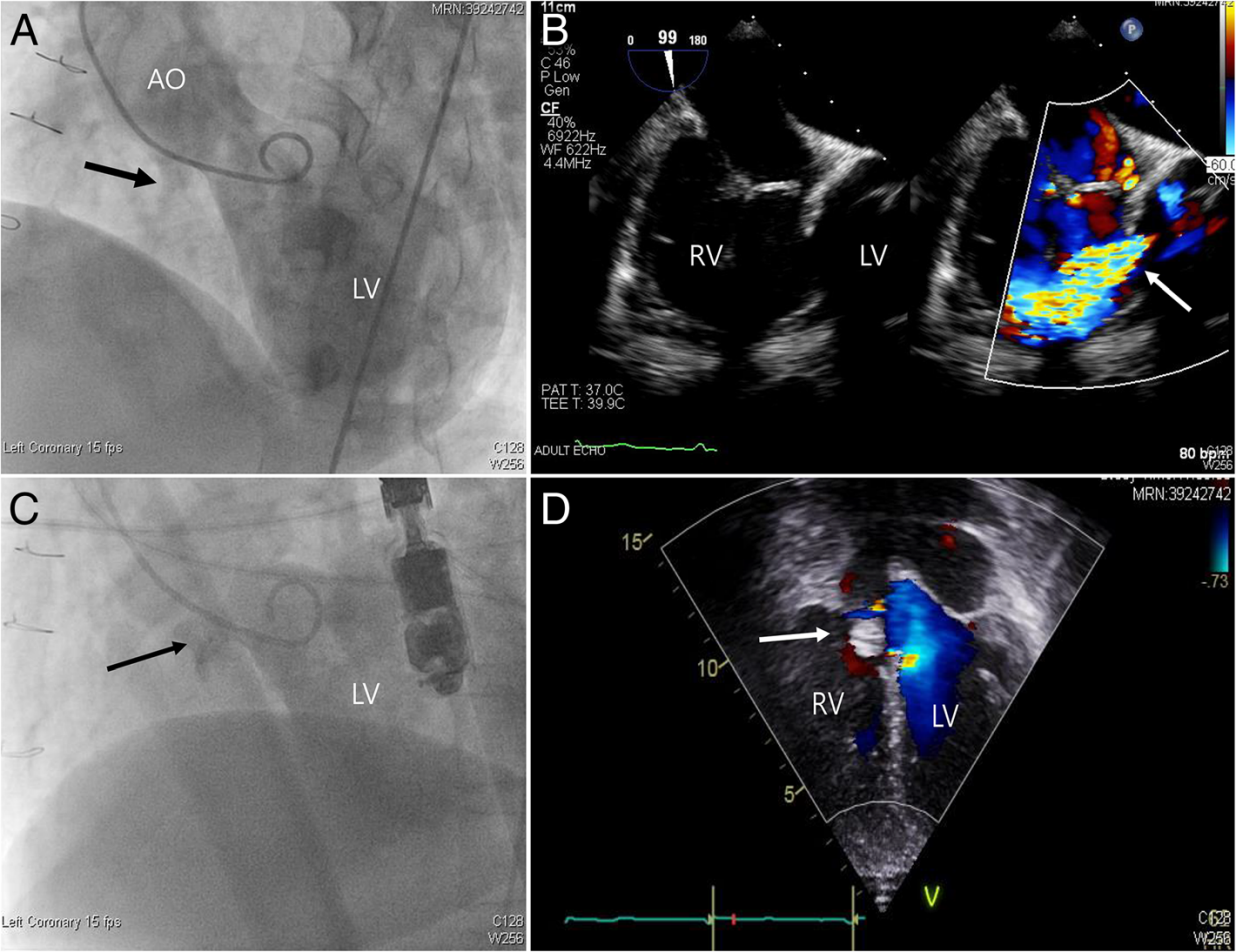
Device closure of residual postopertive VSD with Coccoon VSD occlude device. **a** LV angiography showed residual VSD (black arrow). **b** transesophageal echocardiography and color Doppler revealed residual VSD (white arrow) with significant amount. **c** LV angiography showed successful implantation of the Cocoon device (black arrow) with no significant leakage. **d** transthoracic echocardiography showed successful device closure after the procedure (white arrow). LV, left ventricle, AO, aorta, RV, right ventricle, VSD, ventricular septal defect

**Fig. 2:**
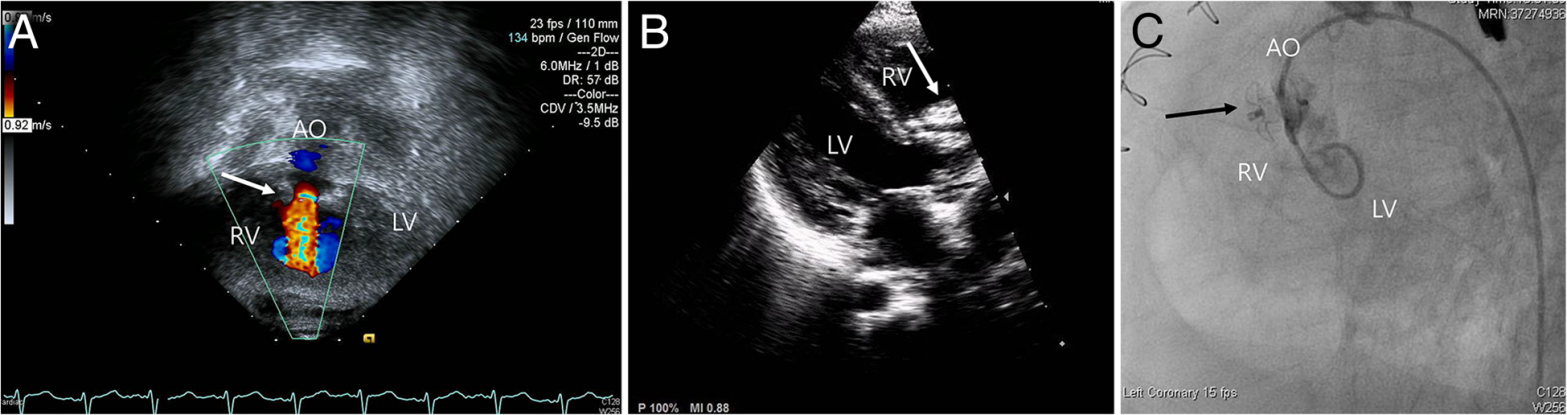
Device closure of VSD fenestration with Coccoon VSD occlude **a**, Transthoracic echocardiography and color Doppler showed VSD through patch fenestration (white arrow). **b** Two dimensional echocardiography showed implanted Coccoon device successfully (white arrow). **c** A good device position (black arrow) and immediate minimal leakage was proven on LV angiography. LV, left ventricle, AO, aorta, RV, right ventricle, VSD, ventricular septal defect

**Fig. 3:**
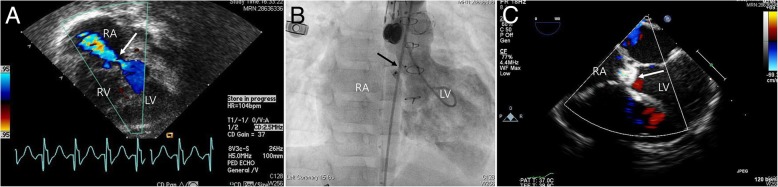
Device closure of Gerbode postoperative VSD with Amplatzer duct occlude I. **a** Gerbode shunt (LV-RA) was shown on transthoracic echocardiography and color Doppler (white arrow). **b** LV angiography showed Amplatzer duct occlude I in a good position after implantation (black arrow). **c** Transthoracic echocardiography showed successful closure of Gerbode shunt with device (white arrow). LV, left ventricle, AO, aorta, RV, right ventricle, RA, right atrium, VSD, ventricular septal defect

VSD closure was performed successfully in all patients. Patient data are summarized in Table 1. The median age and body weight at the time of catheter closure were 17.7 years and 36.4 kg, respectively. The original diagnoses and surgeries were as follows: isolated VSD closure in two patients; tetralogy of Fallot (TOF) total correction; Fallot type double outlet of the right ventricle (DORV) total correction; Rastelli operation for DORV with coarctation**;** and Ross-Konno operation for congenital aortic stenosis. A systolic murmur was audible in all six patients. Pulmonary hypertension was observed in three patients. The reasons for postoperative VSD were residual shunt in three patients (Fig. 1a-d), fenestrated patch in two patients (Fig. 2a-c), and iatrogenic Gerbode shunt in one patient (Fig. 3a-c). The median time between surgery and percutaneous closure was 10.4 years (0.3–33.0 years).

**Table 1:** Basic clinical characteristics of cases

	Sex	Weight (Kg)	Age of operation	Age of device closure	Original CHD/operation	Reasons of residual VSD	Closure indication	Device/size	Combined Procedure	Immediate leak	F/U period (year)	Last echo cardiac
1	M	55.0	47 yr	48.7 yr	TOF/total repair	Fenestration	Qp/Qs 1.9 Mean PA 38 mmHg	Cocoon VSD occlude membranous type/7.5 mm	Nothing	No	2.3	No leak
2	M	84.7	8 yr	33 yr	PM VSD/closure	Leakage	Qp/Qs around 1.5	ADO II/4–4 mm	Nothing	No	3.2	No leak
3	F	11.2	6mo	2.4 yr	DORV (TOF type)/total repair	Iatrogenic (Gerbode shunt)	LV dilatation LPA stenosis	ADO I/5–4 mm	LPA stent	Yes	4.4	Small leak
4	M	7.6	1mo	8mo	Congenital AS/Ross-Konno operation	Iatrogenic	Elevated LVEDP, moderate PS	AVP II/10 mm	PS balloon	No	4.8	No leak,
5	F	53.2	10 yr	43 yr	MO VSD/closure	Leakage	Qp/Qs 1,5 Mean PA 26 mmHg	Cocoon VSD occlude membranous type/7.5 mm	Nothing	Yes	0.7	No leak
6	F	7.0	4mo	7mo	DORV (VSD type) CoA/Norwood and Rastelli operation	Fenestration	Cardiomegaly	Cocoon VSD occlude membranous type/5.5 mm	Nothing	Yes	1.2	No leak

*F/U* follow-up, *TOF* tetralogy of Fallot, *Qp* pulmonary flow, *Qs* systemic flow, *PA* pulmonary artery, *PM* perimembranous, *VSD* ventricular septal defect, *ADO* Amplatzer duct occluder, *ORV* double outlet right ventricular, *LV* left ventricle, *LPA* left pulmonary artery, *AS* aortic stenosis, *LVEDP* left ventricular end-diastolic pressure, *PS* pulmonary stenosis, *AVP* Amplatzer vascular plug, *MO* muscular outlet, *Coa* coarctation of the aorta

We used one device for each patient. We used the following devices: Amplatzer duct occluder (ADO) I; ADO II; Amplatzer vascular plug (AVP) II; or a Cocoon membranous VSD occluder. The potential major complications associated with this procedure include death, device embolization, heart block, new valvular regurgitation, hemolysis requiring blood transfusion, or the need for surgical or percutaneous re-intervention. None of these complications arose in our patients. The immediate results were satisfactory. Small leaks were present in three patients. The median follow-up period was 2.8 years (3.0 months–4.8 years). All of the patients had successful results, except for one patient, who had a residual minimal leak.

## Discussion

Although this study included a very small number of cases, we found that transcatheter closure of postoperative VSD can be performed safely and effectively using various devices. The postoperative VSDs in our series not only resulted from residual leaks, but also from fenestration and Gerbode shunt.

Patients 3 years old and over with hemodynamically significant perimembranous VSD that is adequately separated from the aortic and tricuspid valves are ideal candidates for a transcatheter approach. In cases of a muscular VSD, transcatheter closure is also considered suitable for children weighing ≥5 kg [7]. However, on review of the literature, we did not find any mention of transcatheter closure of postoperative VSDs. It is generally accepted that most postoperative VSDs can be safely closed using such a device. However, the location of the VSD, and the reasons for its development must be considered to identify good candidates for this type of closure. It is sometimes difficult to determine the precise location of postoperative VSDs. Therefore, a comprehensive understanding of the prior surgery is critical. For instance, if the post-operative VSD is located at the center of a patch of a previous VSD (that was closed with fenestration), the risk of aortic valve injury, tricuspid valve injury, or heart block should be minimal. Otherwise, the most important factor is the VSD’s distance from the aortic valve.

Most cases of postoperative VSD result from leakage following VSD closure. Most residual defects < 2 mm in size will close spontaneously, while those > 2 mm will not [7, 8]. Volume overload and infective endocarditis are two reasons that a postoperative VSD requires intervention. We described three patients with leakage of the VSD and evidence of volume overload who were successfully treated.

We also report one patient with a Gerbode defect involving a left ventricular to right atrial communication after DORV total correction. Acquired Gerbode shunts are thought to result from endocarditis, trauma, myocardial infarct, and as a complication of cardiac surgery [9, 10]. The percutaneous closure of Gerbode shunts has previously been described by Song et al. [11] Due to its proximity to the atrioventricular node and the tricuspid valve, close monitoring is necessary both during and after percutaneous closure of a Gerbode shunt.

Two patients in this study had residual VSDs due to fenestrated patch closures. In these circumstances, VSD closure is performed using a fenestrated or valved patch to reduce the risk of right heart decompensation [12, 13]. After postoperative stabilization and further interventional management of hypoplastic or stenotic pulmonary arteries, VSD closure may be necessary at a later date. Transcatheter closure of these VSDs remains challenging. It is particularly difficult due to the extremely flat anatomy of the patch material, and the short distance to the aortic valve [12].

Most patients had evidence of volume overload. However, it was difficult to measure Qp/Qs given the patients’ condition during the procedure. Two patients not only had postoperative VSD, but also pulmonary stenosis. We corrected the pulmonary stenosis and VSD simultaneously, because a VSD shunt might increase in intensity after relief of pulmonary stenosis alone. Therefore, postoperative VSDs should be evaluated in consideration of the patient’s overall cardiac condition.

We used various devices in the closure procedures. The Amplatzer muscular VSD occluders, perimembranous VSD occluders, ADOs, and septal occluders have previously been used to close VSDs percutaneously [14, 15]. We had good results when the postoperative VSD was closed using a cocoon membranous VSD occlude. There were no complications during follow-up in these cases. In general, several complications have been reported after percutaneous closure of a postoperative VSD. These include death, device embolization, heart block, new valvular regurgitation, hemolysis requiring blood transfusion, and the need for subsequent surgical or percutaneous interventions [1–4]. The most serious complication is complete atrioventricular block (cAVB), especially after transcatheter closure of a perimembranous VSD. The incidence of cAVB ranges 0–5.7% [1]. The occurrence of cAVB is related to proximity of the conduction system to the margins of the VSD. Fibrosis or scar formation can occur in the margins of the VSD after a previous operation. Therefore, we suspected that the abovementioned subsequent negative influence of the occluder might decrease, reducing the occurrence of cAVB after closure of postoperative residual VSD [4]. In addition, we used the femoral arteriovenous loop for all cases. However, in some cases, only the femoral vein or artery approach is possible. Therefore, it depends on the location of the VSD and the device. Devices with symmetric retention discs can be implanted through the femoral artery.

Based on our experience, we can recommend transcatheter closure of postoperative VSDs in patients with symptoms of failure. Most postoperative VSDs can be closed using various device types. Transcatheter closure can only be safely performed in patients > 7 kg. In particular, fenestrated VSDs can be safely and effectively closed if the fenestration is no longer necessary. However, surgical closure should be considered if combined lesion also requires surgical repair.

Our study has several limitations. This was a retrospective study based on a small series of cases from a single tertiary center. It also had a short follow-up period. Therefore, the study design itself may have introduced bias. We were unable to measure necessary objective data before and after the intervention, including the Qp/Qs and pulmonary pressures. Therefore, no definitive conclusions should be drawn from our findings.

## Conclusion

Transcatheter closure of postoperative VSD was performed safely and effectively in our sample of patients. Transcatheter closure seems to be an effective alternative treatment option for postoperative VSD. Given the small number of participants in our study, further analysis is required to evaluate its long-term safety and efficacy.

## Data Availability

All data generated or analyzed during this study are included in this published article.

## Abbreviations

ADO: Amplatzer duct occlude
AVP: Amplatzer vascular plug
cAVB: Complete atrioventricular block
DORV: Double outlet of the right ventricle
TEE: Transesophageal echocardiography
TOF: Tetralogy of Fallot
TTE: Transthoracic echocardiography
VSD: Ventricular septal defect
